# Duckweed: Research Meets Applications—2nd Edition

**DOI:** 10.3390/plants15121846

**Published:** 2026-06-15

**Authors:** K. Sowjanya Sree, Klaus-Juergen Appenroth, Viktor Oláh

**Affiliations:** 1 School of Biotechnology, Institute of Science, Banaras Hindu University, Varanasi 221005, India; 2 Matthias Schleiden Institute–Genetics I, Friedrich Schiller University Jena, 07743 Jena, Germany; 3 Department of Botany, Institute of Biology and Ecology, Faculty of Science and Technology, University of Debrecen, 4032 Debrecen, Hungary

## 1. Introduction

**Figure 1:**
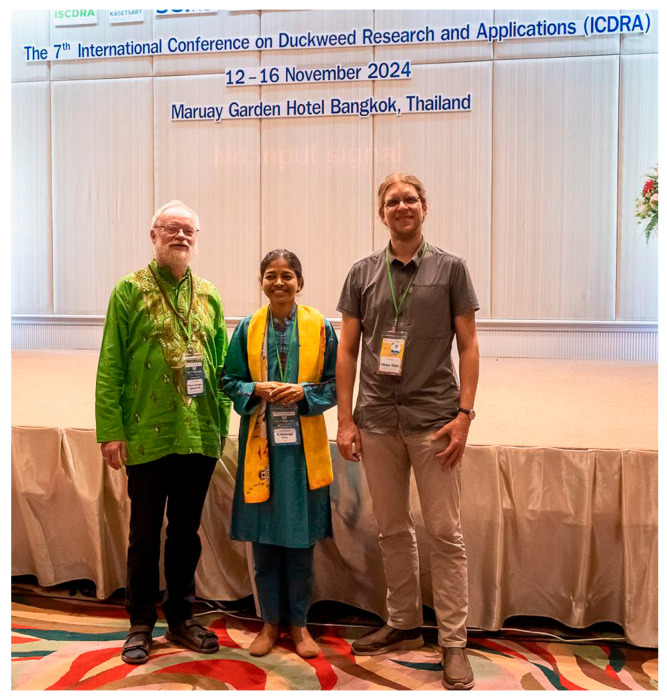
Guest Editors Klaus-Juergen Appenroth (left), K. Sowjanya Sree (middle) and Viktor Oláh (right) of the Special Issue “Duckweed: Research Meets Applications—Second Edition” at the closing ceremony of the 7th International Conference on Duckweed Research and Applications (ICDRA), held in Bangkok, Thailand, 12–16 November 2024.

This second edition of the Special Issue “Duckweed: Research Meets Applications” provides an update on the progress made in recent years in research on and the development of practical applications of duckweeds, tiny aquatic plants also known as water lentils (Lemnaceae Martinov; [1]). These monocots have emerged as model plants for research in various areas of plant biology [2,3]. This edition comprises 12 original research articles, one review article, one perspective, and one conference report, several of which showcase national and international scientific and research cooperation amongst various groups of researchers worldwide. The 7th International Conference on Duckweed Research and Applications (7th ICDRA) held in Thailand from 12 to 16 November 2024 not only highlighted the progress made in the duckweed research arena but also the boom in the practical applications of duckweeds, especially regarding their use for human nutrition. A glimpse of the advances presented there is reported in the conference report (**7th International Conference on Duckweed Research and Applications: Depicting an Era of Advancing Research Translation Toward Practical Applications**) by Appenroth et al. [4] (Figure 1).

The present editorial provides an overview of the articles published in this Special Issue, which are broadly categorized into sections on the (1) physiology, (2) genetics and (3) applications of duckweeds. The titles of the published articles are highlighted in bold for easy reference.

## 2. Duckweed Physiology

Duckweeds have helped in many past discoveries in plant physiology by acting as models. Krzeszowiec and Gabryś [5] presented the latest example of this traditional role by sharing new insights into the role of glutamate receptor-like (GLR) channels in plants (**GLR Channels Are Involved in the Mechanism of Chloroplast Avoidance Response in *Lemna trisulca***). They evidenced that NMDA (N-methyl-d-aspartate)-activated GLR channels participated in controlling light-dependent chloroplast movements of *Lemna trisulca* fronds. These channels, besides being proven to be pH-dependent, also showed functional separation from other GLR channels that did not play a role in the chloroplast avoidance response.

Ruamsin et al. [6] compared the sensitivity of two duckweed species to an environmentally relevant heavy metal, mercury (**Comparative Physiological Responses of *Lemna aequinoctialis* and *Spirodela polyrhiza* to Mercury Stress: Implications for Biomonitoring and Phytoremediation**). By analyzing the growth and physiological responses of *Lemna aequinoctialis* and *Spirodela polyrhiza*, they concluded that the former species was applicable in the biomonitoring of Hg, while the latter showed higher tolerance and was thus a more suitable candidate for bioremediation.

Despite clonality, even fronds of the same colony may provide different responses to environmental stimuli as a function of their age [7,8]. In this Special Issue, two papers addressed this emerging topic. Peršić et al. [9] revealed that *S. polyrhiza* fronds displayed age-specific adjustments to sulfur limitation (**Age-Specific Physiological Adjustments of *Spirodela polyrhiza* to Sulfur Deficiency**): while mother fronds relied on their internal reserves, granddaughter fronds maintained homeostasis by dynamically regulating light absorption, energy distribution and carbon sinks. In contrast, daughter fronds, which normally possessed the highest photosynthetic capacity, suffered the strongest decline under sulfur deprivation. Xuan et al. [10] tracked the photosynthetic adjustment of *Lemna gibba* fronds to different light intensities (***Lemna gibba* Clones Show Differences in Phenotypic Responses to the Light Environment**) and found that this acclimation involved morphological, physiological and biochemical changes and happened during the early phases of frond ontogeny. In addition to frond maturation, genotype also had an effect on light acclimation, resulting in partially diverging strategies in various clones.

In addition to the case studies, a review paper by Ziegler [11] also addressed duckweed fronds, specifically a very special group of them: resting fronds (**The Developmental Cycle of *Spirodela polyrhiza* Turions: A Model for Turion-Based Duckweed Overwintering?**). This study provided a thorough overview of the known resting frond types in the Lemnaceae family and synthesized the state-of-the-art knowledge on the formation, dormancy, activation and germination of *S. polyrhiza* turions. After elegantly summarizing almost 80 years of research, Ziegler [11] argued in his conclusion that these findings on *S. polyrhiza* turions cannot be generalized to resting fronds of other duckweed species, though they can serve as the basis for further research.

## 3. Duckweed Genetics

Variations in the physiological properties of duckweeds based on their genetic diversity are not limited to the species level; rather, they are also highly pronounced at the clone level [12]. The perspective article by Morello et al. [13] underlined the importance of duckweed clone collections, specifically highlighting the pioneering efforts made by late Elias Landolt in this direction (**Exploring Duckweed Diversity at the Dawn of Its Cultivation Era: The Invaluable Legacy of the Landolt Collection**). The need for improving duckweed breeding efforts is also highlighted in this article, as a complement to the growing interest and progress in realizing practical applications of duckweeds. In line with this, a research article by Senayai et al. [14] assessed the genetic diversity of duckweeds collected from across Thailand (**Genetic and Morphological Variation Among Populations of Duckweed Species in Thailand**). They assessed the level of intraspecific genetic variation among clones of duckweeds collected from four different species along 26 sites in Thailand. Such studies highlight the importance of investigating duckweed genetic diversity for the use of plants available locally in different practical applications. A specific example of the analysis of duckweeds at a molecular level showcasing their use in producing starch-rich duckweed biomass was reported by Fang et al. [15], where they investigated the metabolomics and transcriptomic profile of duckweeds exposed to short-term nutrient stress, which suggested an increase in starch synthesis and a decrease in starch degradation, leading to starch accumulation under nutrient stress (**Transcriptomic and Metabolic Analysis Reveal Potential Mechanism of Starch Accumulation in *Spirodela polyrhiza* Under Nutrient Stress**).

## 4. Applications of Duckweed

In six original contributions, a broad range of practical applications of duckweeds were presented. Some of these are related to using and/or cleaning wastewater. In many European areas with intensive livestock farming, waste from different animals is a serious threat to the environment and at the same time a valuable resource that is still wasted at present. Lambert et al. [16] used pig manure as a medium for the cultivation of the hybrid *Lemna x japonica* during a growing season in Belgium (**A Pilot-Scale Evaluation of Duckweed Cultivation for Pig Manure Treatment and Feed Production**). In a diluted liquid fraction and nitrification–denitrification effluent, duckweed biomass with a high protein content was produced simultaneously using the system for efficient N and P removal. This biomass could be used for feeding not only broilers but also other farm animals.

A group in Cork, Ireland, continued to investigate the cultivation of *L. minor* on wastewater, comparing cultivation on half-strength Hutner’s medium with the liquid fractions of anaerobically digested pig slurry [17]. They discovered that the light regime required depends on the nutrient medium used (**Varied Growth Media Necessitate Different Light Regimes for Indoor Duckweed Cultivation**). Under some conditions, the photoperiod, rather than the light intensity, was the most critical factor, whereas, under other cultivation conditions, it was the other way around. Also, dairy-soiled water, produced mainly during the milking of cows (**Optimisation of Dairy Soiled Water as a Novel Duckweed Growth Medium**), was investigated as a source of plant fertilization. The problem here is again the huge volume and the high variability depending on farming practices. Redmond et al. [18] reported progress in this procedure and identified pH as a key parameter for the optimization of growth rates and the protein content of the *L. minor* biomass within a circular economy.

In the last few years, several papers have been published analyzing the opportunity to cultivate duckweeds under vertical cultivation in controlled environments. A group from the University of Applied Sciences Osnabrueck, Germany, modeled the growth of *L. minor* under different plant density and light intensity conditions [19] (**Modeling Growth Dynamics of *Lemna minor*: Process Optimization considering the Influence of Plant Density and Light Intensity**). This model can be used to predict optimal cultivation conditions, e.g., concerning harvest quantities.

Han et al. [20] investigated the toxic effects of metformin, a medical drug used to treat diabetes, at the physiological and transcriptional level (**The Toxicity Effects of Metformin and the Bioremediation of Metformin in Aquatic Plant Duckweed**). Interestingly, *Lemna turionifera* showed a high accumulation level (0.128 mg/g FW) and, after 10 days of cultivation, the metformin concentration in the wastewater was down to practically zero.

The effect of the co-cultivation of duckweed and rice in paddy fields, mainly via the spontaneous outbreak of duckweed populations, was investigated by Zhao et al. [21] (**Duckweed’s Effects on Rice Yield and Quality Varied with Fertilizer Applications**). There is a clear influence of duckweed on rice yield, starch quality and palatability, but these effects depend very strongly on the fertilization regime.

## 5. Further Outlook

The present Special Issue “Duckweed: Research meets Applications” demonstrates the enormous progress made in research and applications in the field of duckweed in the last few years. In the plenary lecture of the 7th International Conference on Duckweed Research and Applications in Bangkok, Thailand, the following fields were mentioned that deserve more attention as key points for progress in the future [22]:

- Large-scale industrial production of duckweed biomass;
- Large-scale industrial production of protein or starch from duckweed;
- Taxonomy of duckweeds;
- Further genome sequencing;
- Genetic transformation;
- Single-cell transcriptomics—for duckweed development;
- Interaction of duckweeds with microorganisms;
- Wastewater phytoremediation;
- Duckweeds for human nutrition.

The 8th International Conference on Duckweed Research and Applications will take place in Portici, Naples, Italy from 28 September to 2 October 2026 under the tagline “**Model plant and novel crop, the thousand faces of duckweed**”. In order to track the progress made in the field of duckweed research and applications, a third edition of this Special Issue will soon be open.

** Dedicated to Professor Helmut Augsten (born 8 May 1928) on the occasion of his 98th birthday, who initiated duckweed research and the duckweed stock collection at the University of Jena, Germany, 60 years ago, which is still being actively carried out to this day by one of the authors, KJA.
